# Gain in cellular organization of inflammatory breast cancer: A 3D *in vitro *model that mimics the *in vivo *metastasis

**DOI:** 10.1186/1471-2407-9-462

**Published:** 2009-12-23

**Authors:** Jorge Morales, Mary L Alpaugh

**Affiliations:** 1 Department of Biology, City University of New York, The City College of New York 138th and Convent Avenue, New York, NY 10031, USA

## Abstract

**Background:**

The initial step of metastasis in carcinomas, often referred to as the epithelial-mesenchymal transition (EMT), occurs via the loss of adherens junctions (e.g. cadherins) by the tumor embolus. This leads to a subsequent loss of cell polarity and cellular differentiation and organization, enabling cells of the embolus to become motile and invasive. However highly malignant inflammatory breast cancer (IBC) over-expresses E-cadherin. The human xenograft model of IBC (MARY-X), like IBC, displays the signature phenotype of an exaggerated degree of lymphovascular invasion (LVI) *in situ *by tumor emboli. An intact E-cadherin/α, β-catenin axis mediates the tight, compact clump of cells found both *in vitro *and *in vivo *as spheroids and tumor emboli, respectively.

**Methods:**

Using electron microscopy and focused ion beam milling to acquire *in situ *sections, we performed ultrastructural analysis of both an IBC and non-IBC, E-cadherin positive cell line to determine if retention of this adhesion molecule contributed to cellular organization.

**Results:**

Here we report through ultrastructural analysis that IBC exhibits a high degree of cellular organization with polar elements such as apical/lateral positioning of E-cadherin, apical surface microvilli, and tortuous lumen-like (canalis) structures. In contrast, agarose-induced spheroids of MCF-7, a weakly invasive E-cadherin positive breast carcinoma cell line, do not exhibit ultrastructural polar features.

**Conclusions:**

This study has determined that the highly metastatic IBC with an exaggerated malignant phenotype challenges conventional wisdom in that instead of displaying a loss of cellular organization, IBC acquires a highly structured architecture.

These findings suggest that the metastatic efficiency might be linked to the formation and maintenance of these architectural features. The comparative architectural features of both the spheroid and embolus of MARY-X provide an *in vitro *model with tractable *in vivo *applications.

## Background

Normal epithelial tissue has a distinct architecture. The organization and maintenance of this tissue architecture is mediated by cell-cell adhesion junctions [1]. The most notable of the adhesion junctions is the adherens junction (AJ). The AJs are critical for the formation of a polarized epithelial sheet composed of two structurally distinguishable surfaces, namely the apical and basal [1-3]. The transmembrane protein E-cadherin forms AJs and binds to the family of catenins (e.g. α-catenin, β-catenin, p120) on the cytoplasmic side of the plasma membrane [4,5]. This intact E-cadherin α/β-catenin axis regulates cytoskeleton dynamics [2]. Nuclear translocation of β-catenin upon cleavage of a formerly intact axis alters gene transcription [4]. Positioned directly adjacent to the AJ is the tight junction (TJ). In glandular epithelium the TJs are lumenally-located (apical face) in relationship to the AJs. These junctions are predominantly composed of claudin proteins and are responsible for regulating ion permeability [1,6]. More basally-located are the desmosomes (DMs) and these junctions provide resistance to shear stress of epithelial tissue and are composed of desmosomal cadherins (desmocollins, desmogleins) [1,7]. The desmosomal cadherins are linked to intermediate filaments of plaque proteins (desmoplakin, plakoglobin and plakophillin) in the cytoplasm [7]. The hallmark structure of a polarized epithelial sheet is the tripartite complex composed of a TJ, AJ and DM. This structural complex is observed in electron micrographs and is located at the apex (apical/lateral) of adjacent cells [2,8]. In normal epithelial tissue polarity results in an asymmetric distribution of protein receptors/transporters, signaling complexes, ion channels and lipids between two surfaces, the apical and basolateral [1]. It is the maintenance of the architectural integrity and function of the two surfaces which traditionally distinguish normal from aberrant cells.

Most cancers arise from epithelial cells and the initial step in metastatic progression is reduced intercellular adhesion [9,10]. This is primarily associated with the loss of E-cadherin function with subsequent loss of cell polarity, epithelial differentiation and organization [11-13]. With the loss of positional cues the cancer cells are relieved of contact inhibition of growth and become more motile and invasive [10]. A loss of E-cadherin results in a loss in all epithelial features and is sufficient to accelerate the adenoma-to-carcinoma transition in mouse tumor models, indicating that loss of E-cadherin may be a rate-limiting step in tumor progression [14].

Reduced expression of E-cadherin with diminution of cell-cell junctions is generally accepted as a malignancy indicator [15]. Paradoxically, human inflammatory breast cancer (IBC), a highly metastatic carcinoma, over-expresses E-cadherin [16-18]. The human xenograft model of IBC (MARY-X), like IBC, displays the signature phenotype of an exaggerated degree of lymphovascular invasion (LVI) *in situ *by tumor emboli [19]. MARY-X also exhibits a 10 - 20 fold over-expression of an intact E-cadherin/α, β- catenin axis [19]. This over-expressed highly functional adhesion molecule mediates the tight, compact clump of cells found both *in vitro *as spheroids and *in vivo *as tumor emboli [20,21]. Compaction due to E-cadherin confers resistance to apoptosis [22]. In MARY-X, disruption of the intact axis by Ca^++ ^depletion, E-cadherin antibody, glycan modification of MUC1 and trypsin proteolysis results in the total dissolution of the *in vitro *spheroids followed by apoptosis, suggesting that over-expressed E-cadherin/α, β-catenin axis plays an important role in the survival of highly metastatic IBC [20-22].

In this study, using the human xenograft model of IBC (MARY-X) we show that malignant IBC displays architectural features or a gain in cellular organization that is not typically found in aggressive carcinomas. This architecture, found both *in vitro *and *in vivo*, exhibits intact tight junctions, adherens junctions and microvilli coating of the apical surface of lumen-like structures (canalis), which are evenly distributed throughout the MARY-X spheroid and tumor cell embolus. This 3D *in vitro *model that truly mimics the *in vivo *physiological/pathological conditions provides tractable information concerning structural architecture and metastatic behavior of the *in vivo *tumor cell embolus.

## Methods

### MARY-X xenograft and *in vitro *spheroids

MARY-X was established from a patient with inflammatory breast cancer (IBC) [19]. *In vivo*, MARY-X recapitulates the human IBC phenotype of extensive lymphovascular invasion of the tumor cell emboli. The IBC spheroids are a cellular derivative (i.e. primary cell line) of MARY-X primary tumor explants. Upon mincing the tumor cells are released into the media as sheets of cells and single cells. The tumor cells form tight, compact clumps or aggregates of cells termed "MARY-X spheroids". These spheroids can be further purified or partitioned from the cellular debris by employing cell strainers of varying pore sizes (e.g. 40, 70 and 100 μm; BD Biosciences) [21]. The resultant preparation is 100% human IBC cells (termed MARY-X spheroids) which can be maintained in culture for periods up to three months.

The MARY-X spheroids when injected into severe-combined immune deficient (SCID) mice, form complex primary tumors (and distant lung metastases) where the tumor cell emboli are found nestled within the murine lymphatics and blood vessels (i.e. lymphovascular invasion) [19]. The tumor is composed of a 30% murine component (surrounding stroma, lymphatic vessels and blood vessels) and 70% human inflammatory breast cancer cell component (tumor cell emboli) [19].

MCF-7 (American Type Culture Collection, Rockville, MD) spheroids were established by plating MCF-7 cells on a 1% agarose-coated tissue culture plate.

All cells were maintained in minimal essential medium (MEM) containing 10% fetal bovine serum and antibiotics (100 U/ml penicillin and 100 μg/ml streptomycin) at 37°C in an air-5% CO_2 _atmosphere at constant humidity.

All experiments were performed in compliance with the Memorial Sloan-Kettering Cancer Center Animal Care and Use Program (Protocol Number 06-04-006).

### *In vitro *Dissolution Studies

The MARY-X spheroids underwent full dissolution by either calcium depletion or the adhesion-blocking E-cadherin antibody (Invitrogen, clone HECD-1) as previously reported [22]. Cytospin preparations were obtained for dissolute spheroids.

### Transmission Electron Microscopy

MARY-X spheroids, tumor emboli and MCF-7 agarose-induced spheroids were fixed for 2-24 hours at 4°C in 3% Glutaraldehyde/0.1 M sodium cacodylate buffer (pH 7.2) and post-fixed for 45 min in buffered 1% osmium tetroxide. The samples were dehydrated in a series of ethanol solutions followed by a propylene oxide rinse and infiltrated with Embed 812 resin. Thin sections (120 nm) were cut with a diamond knife on a LKB microtome and collected on nickel grids (EMS: G300H-Ni). Sections were stained in 4% uranyl acetate for 1-2 hours followed by 0.6% lead citrate for 20 minutes. Sections were viewed on a Zeiss EM902 transmission electron microscope and images were captured digitally using a Mega View III CCD camera with a pixel resolution of 1.3 Megapixels. Analysis of spheroid preparations was performed on spheroid pellets (5,000 - 10,000 spheroids/pellet). TEM viewing was performed on 20 - 30 randomly chosen fields of multiple spheroid pellet and tumor emboli preparations.

### Scanning Electron Microscopy

MARY-X spheroids, tumor emboli and MCF-7 agarose-induced spheroids were fixed and post-fixed as described in the TEM protocol above, dehydrated in a series of ethanol solutions and dried using a critical point dryer (Balzer CPD-030). The dried samples were coated with a 2 -3 nanometers of gold using a Denton Desk II sputter coater. Observations were made using a scanning electron microscope (Supra 55 VP or Quanta 200 3D). Spheroid pellet preparations (5,000 - 10,000 spheroids/pellet) are mounted (scattered) onto pins and sputter-coated as described. Observations were performed on 10 - 20 individual spheroids of multiple preparations.

### Scanning Electron Microscopy/Focused Ion Beam Milling

Milling of the specimens was performed using a Quanta 200 3D dual beam SEM-FIB. *In situ *sections (i.e. milling) were achieved with an initial blunt section at a current of 20 nA, followed by decreasing currents (20 - 3nA) for polished sections. Images were captured at a 1 Megapixel resolution.

### Immunofluorescence

The antibodies used for immunofluorescence included E-cadherin (BD Transduction labs; catalogue #610181) and β-catenin (Sigma; catalogue #C 2206). The monoclonal antibody for E-cadherin recognized the intracellular domain. Immunofluorescence was observed using an upright Leica TCS SP2 AOBS 1- and 2-photon laser scanning confocal microscope.

## Results and Discussion

### Polar Architectural Features of MARY-X

**Figure 1:**
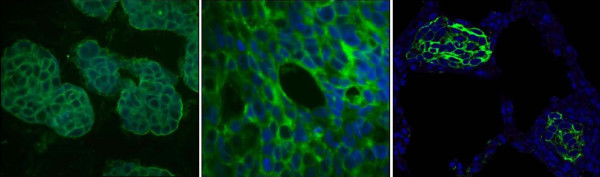
**E-cadherin expression of MARY-X: **The MARY-X spheroids (left panel), tumor emboli of a primary tumor (middle panel) and emboli of pulmonary metastases (right panel) display strong membrane-located E-cadherin staining (40× magnification). Nuclei of all sections were stained with 4',6-diamidino-2-phenlylindole (DAPI).

Architectural integrity or structural organization is known to be disrupted following loss of cell-cell adhesion during pathogenesis (i.e. malignant progression) of epithelial tumors [11,13]. However highly malignant inflammatory breast cancer (IBC) over-expresses E-cadherin. The human xenograft model of IBC (MARY-X), like IBC, displays the signature phenotype of an exaggerated degree of lymphovascular invasion (LVI) *in situ *by tumor emboli and also over-expresses E-cadherin. This over-expression of E-cadherin by MARY-X is maintained throughout the metastatic progression (Figure 1).

**Figure 2:**
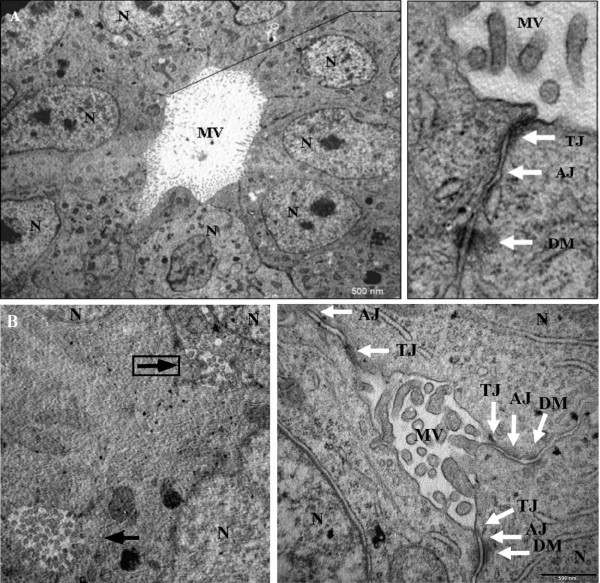
**TEM of MARY-X spheroids: **(A; left panel, B; left panel, arrows) Canalis with extensive microvilli (MV) are found on the interior of several adjoining cells (N; nucleus). (A, B; right panels, note that right panel of B correlates with boxed arrow on the left panel) Found at the apex (apical/lateral) of adjoining cells within the canalis are tight junctions, adherens junctions and desmosomes (TJ; arrow, AJ; arrow and DM; arrow).

Transmission electron microscopic (TEM) analysis was performed to investigate the paradox of E-cadherin over-expression in the MARY-X spheroid and whether it extended beyond the molecular function (e.g. compaction and resistance to induction of apoptosis, as previously reported) and contributed to cellular organization. Apparent upon ultrastructural analysis were several lumen-like structures (canalis) formed by adjoining cells located in the inner regions of the MARY-X spheroid (Figure 2A and 2B). Consistent with an apical surface of polar epithelial cells, the surface of the canalis was coated with microvilli (Figure 2A and 2B). Further electron microscopic evaluation under higher magnification of the apex of adjacent cells revealed apical/lateral positioning of AJs (Figure 2A and 2B; right panels). Also present, was the more lumenally-located TJ (referred to also as zonula occludens) and the basally-localized DM (Figure 2A and 2B; right panels). This tripartite complex, TJ, AJ and DM, of the MARY-X spheroid authenticates polarity with the distinct feature of establishing two separate plasma membrane domains, the apical and basolateral surface. These architectural features were also observed in the TEM analysis of the MARY-X tumor cell emboli (data not shown). Initial electron microscopic analysis therefore suggests that the over-expression of the intact E-cadherin/α, β-catenin axis extends beyond molecular function and displays a first order level of structural organization uncommon in a carcinoma with a malignant phenotype. To the best of our knowledge such structural features have not been previously reported in spontaneously forming *in vitro *spheroids.

**Figure 3:**
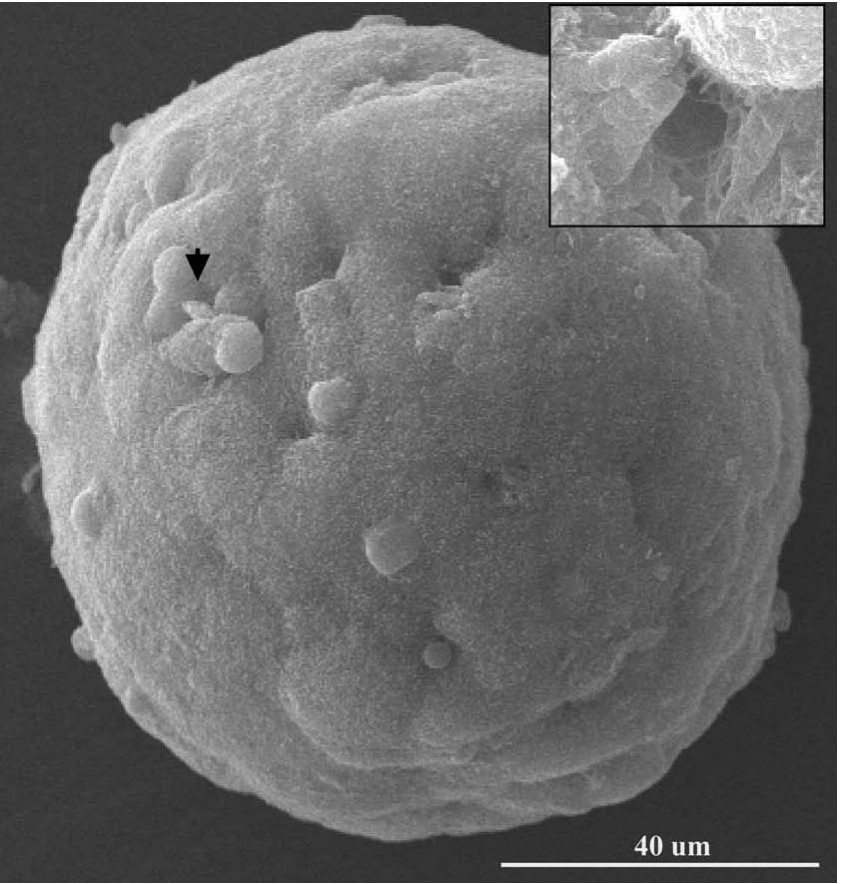
**SEM of MARY-X Spheroid: **SEM analysis reveals multiple "dimple-like" features (e.g. arrow) amidst short microvilli on the surface of the spheroid. Magnification (inset) shows a canal orifice.

**Figure 4:**
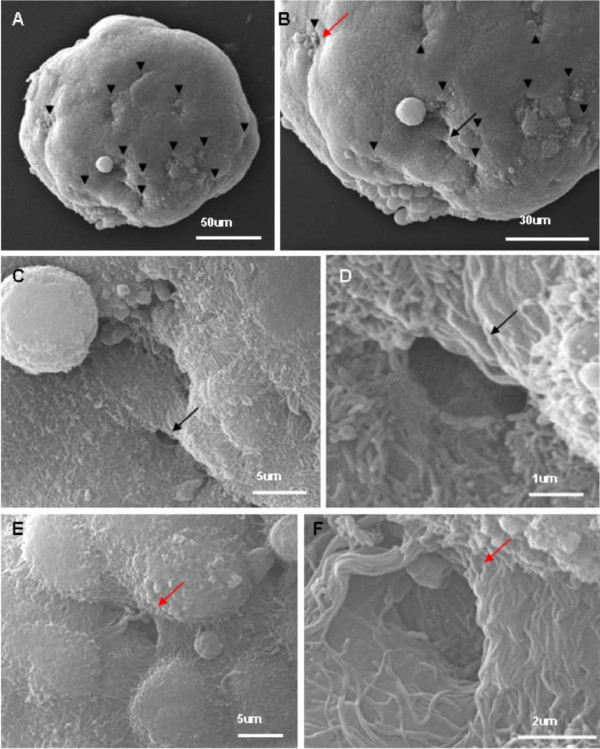
**SEM of MARY-X Spheroid and lumen orifices: **(A and B) Multiple clusters of canalis orifices seen as "dimples" on the surface of the spheroid (black arrowheads and black and red arrows; magnified in C & E). (C and E) Short microvilli coat the surface of the spheroid. (D and F) Magnification of the "dimples" i.e. lumen orifices shows dense, long microvilli lining the exterior portion.

**Figure 5:**
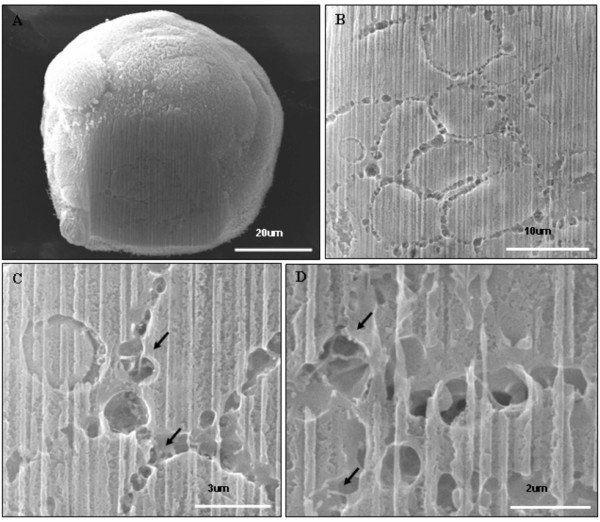
**SEM/FIB milling of MARY-X spheroid: **(A and B) FIB milling of the MARY-X spheroid shows a network of canalis evenly distributed through out the spheroid. (C and D) Microvilli are seen within the canalis (arrows).

The canalis found upon ultrastructural analysis of MARY-X, presented compelling evidence for the existence of a hierarchy of structural architecture within the multicellular spheroid. Given the two-dimensional (2D) nature of the electron micrographs, the images of canalis could represent a series of blind diverticulum or cavities within the spheroid. Therefore to further investigate, tomography of individual MARY-X spheroids (70 μm-200 μm in diameter), was analyzed using SEM (Figure 3). The outer surface of the spheroid is covered extensively with short microvilli. Clusters of dimple-like structures can be seen throughout the spheroid surface (Figure 3; arrow and Figure 4A and 4B; arrow heads). When explored further under higher magnification, these dimple-like structures (Figure 3; inset and Figure 4C-F; red and black arrows) were found to be orifices covered with long, dense, fibrous microvilli (Figure 4D and 4F). While maintaining the three-dimensional (3D) context, the spheroid was then sectioned using the integrated scanning electron microscopy/focused ion beam (SEM/FIB) milling technology. Using the FIB, sections (i.e. milling) were produced *in situ *of the spheroid (Figure 5). The canalis were found to originate as orifices on the surface and extend deep within the interior of the spheroid (Figure 5C-D). The canalis appear to be tortuous and highly bifurcating (Figure 5B). Although not as apparent as was seen on the spheroid surface and orifice, the interior canalis do retain a coating of microvilli (Figure 5C and 5D). Compatible with the TEM findings, the tortuous canalis are formed by the polar assembly of the multicellular spheroid and represent a second order level of structural organization, confirming an architectural hierarchy within the malignant carcinoma, IBC.

**Figure 6:**
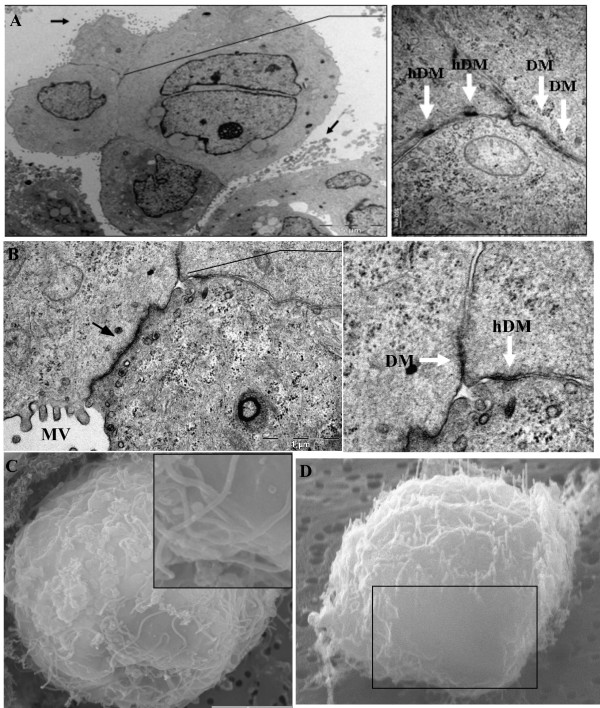
**TEM and SEM of MCF-7 spheroids: **(A, B) TEM analysis reveals MCF-7 spheroids with discontinuous microvilli (arrows) on the surface of the spheroid. (A, B; right panels) Adjoining cells of MCF-7 spheroids form both desmosomes and hemidesmosomes (DM, arrow & hDM, arrow) but no tripartite complex. (C) SEM analysis of agarose-induced MCF-7 spheroids showed discontinuous surface microvilli and no surface orifices (inset). (D) In situ sections (box) revealed a smooth cellular cross section with no lumen-like structures.

E-cadherin contributes to the organized assembly of IBC, however, to determine whether E-cadherin-expression alone or a combined innate predisposition of IBC, contributed to a gain in cellular organization, the ultrastructure of the non-IBC breast carcinoma, MCF-7, was examined. MCF-7 is a weakly invasive non-IBC cell line that like MARY-X expresses a significant level of E-cadherin as reported earlier [21]. The MCF-7 cell line can be induced to form spheroids in culture when attachment as a monolayer is prohibited (i.e. 1% agarose-coated plates). This method of inducing 3-dimensional multicellular spheroids is commonly used to test therapeutic efficacy because these artificial multicellular spheroids more closely resemble the *in vivo *metastasis (i.e. compact clump of cells) [23-26]. When examined by TEM these E-cadherin-expressing spheroids did not display polar elements as seen in MARY-X spheroids (Figure 6). Adjoining cells did not form tripartite complexes but rather continuous junctions predominantly composed of tight junctions (TJs) and/or desmosomes (DM) and hemi-desmosomes (hDM) (Figure 6B; left panel) at what would constitute the apex of the apical/lateral surface. Internal regions of the MCF-7 spheroid revealed similar findings where adjacent cells formed junctions predominantly of DMs and hDMs (Figure 6A and 6B; right panels). Most significantly, MCF-7 ultrastructure did not display internal canalis (Figure 6A and 6B; right panels). The overall surface of the MCF-7 spheroid exhibited discontinuous spheroid surface microvilli (Figure 6A; left panel, arrows). A previous ultrastructural study of agarose-induced MCF-7 spheroids reported similar results with cell-cell contacts predominantly forming tight junctions and desmosomes with no tripartite complex [27]. Scanning electron microscopic (SEM) and focused ion beam (FIB) milling analysis was performed on the agarose-induced MCF-7 spheroids and further confirmed the absence of any level of cellular organization i.e. surface orifices, lumen-like structures (Figure 6C and 6D). The difference in the expression level of E-cadherin and the fact that the MCF-7 cell line does not spontaneously form spheroids *in vitro *may partially explain the absence of cellular organization of the agarose-induced MCF-7 spheroids.

**Figure 7:**
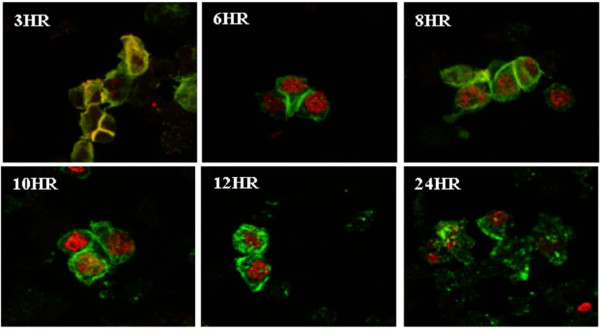
**Nuclear translocation of E-cadherin upon disruption of intact axis: **(Red channel; E-cadherin 1° antibody conjugated to 568 nm fluorophore, Green channel; β-catenin 1° antibody conjugated to a 488 nm fluorophore). (3 HR) Co-localization of E-cadherin and β-catenin at the plasma membrane is seen. (6 HR 24 HR) Prolonged disruption results in E-cadherin nuclear translocation. (10 HR 24 HR) Membrane degeneration is observed.

It is known that cleaving the intact E-cadherin/α, β-catenin axis induces a cascade of signaling events culminating in β-catenin nuclear translocation and alteration of gene transcription [5]. To assess the consequences of disruption of the intact axis in the MARY-X spheroids, dissolution upon Ca^++^ depletion was performed. At time points 3, 6, 8, 10, 12, and 24 hours, aliquots of the dissolute spheroids were removed. Cytospin preparations of the aliquots were double-labeled with a β-catenin antibody and an E-cadherin antibody specific to the cytoplasmic domain of this molecule. At 3 hours of dissolution both β-catenin and E-cadherin co-localize at the plasma membrane, representing an intact axis (Figure 7; 3 HR). Surprisingly, by 6 hours, while β-catenin continued to be positioned at the plasma membrane, E-cadherin was translocated to the nucleus (Figure 7; 6 HR). This appearance was maintained for up to 24 hours (Figure 7; 3 HR-24 HR). From 10 to 24 hours, plasma membrane degeneration was observed. Interestingly, the time scale of E-cadherin nuclear translocation overlapped with the induction of apoptosis in dissolute spheroids from previously reported data, which was also achieved in approximately 6 hours [22]. The nuclear translocation of the cytoplasmic domain of E-cadherin upon dissolution of the formerly intact MARY-X spheroids presents a novel function of E-cadherin. E-cadherin translocation to the nucleus was also observed when disruption of the intact axis was achieved using an E-cadherin antibody (clone HECD) specific for the extracellular domain of the transmembrane protein (data not shown), proving that translocation is not due to a calcium-dependent signaling mechanism. Nuclear translocation of E-cadherin within MCF-7 cells has also been reported in a previous study with a possible role in suppression of drug-induced apoptosis [28]. Albeit the level of E-cadherin is lower in MCF-7 than in MARY-X spheroids, we have found apoptotic response and nuclear translocation of the cytoplasmic domain of E-cadherin upon disruption of molecular function of E-cadherin to be almost equal [22,28]. However, ultrastructural analysis of MCF-7 agarose-induced spheroids was found to be very different. Pursuit of the molecular function of E-cadherin once in the nucleus of MARY-X, although unexpected and interesting, is beyond the scope of this paper.

The information that malignant IBC retains structural integrity gives exciting insight into the possibility that metastatic efficiency in some carcinomas is independent of "loss of function" i.e. loss of cellular differentiation accompanied by higher mobility and invasiveness resulting from reduced intercellular adhesion [11,13]. According to traditional definition, architectural integrity is disrupted during pathogenesis of epithelial tumors and pathological assessment is based predominantly on microscopic evaluation of (loss of) epithelial cell features [11,29]. Histological patterns, such as the loss of E-cadherin expression in carcinomas is the most widely accepted malignant and prognostic indicator in diagnosis of the disease [15]. However, ultrastructural features cannot be seen in these typical evaluations and "gain in function" or structural organization has not been explored with respect to possible clinical outcomes.

### The Polar Architectural Features of the MARY-X *In Vitro *Spheroid Mimic the *In Vivo *Metastasis

**Figure 8:**
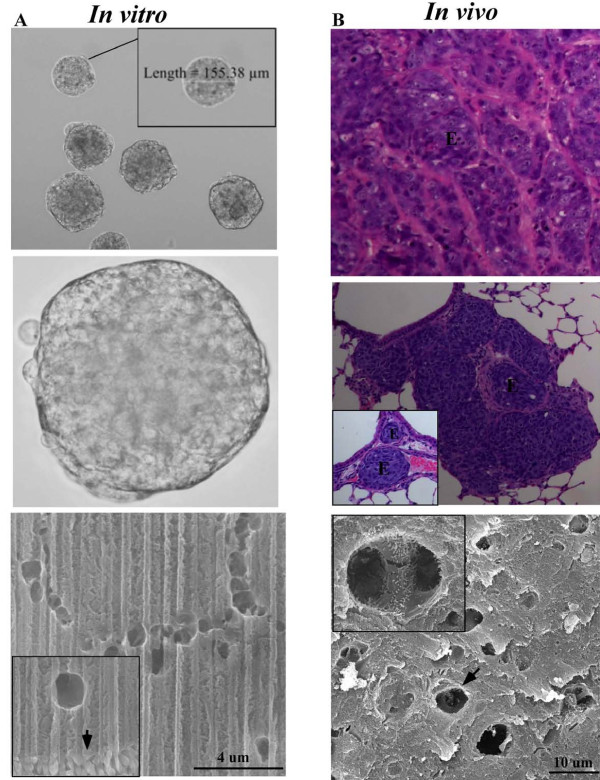
**Spontaneously formed *in vitro *MARY-X spheroids mimic the *in vivo *metastasis: **(A; top and middle panels) *In vitro*, MARY-X forms a compact 3D multicellular structure in suspension, ranging in size from as small as 10 μm to as large as 400 μm in diameter (10× and 40× magnification, respectively). (A; bottom panel) FIB milling of the MARY-X spheroid displays extensive canalis structures throughout the MARY-X spheroid. Extensive surface microvilli (inset, arrow) adjacent to a canal as displayed in the FIB section (B; top and middle panels) A cross-section of a MARY-X primary tumor and murine lung metastases shows compact, multicellular MARY-X lymphovascular emboli (E) (H & E stained, 40× and 20× magnification). The pulmonary metastasis may exist as a simple single distinct lymphovascular embolus (middle panel; inset, E) or a more complex metastasis where the lymphovascular emboli are nestled within the murine surrounding stroma (middle panel, E). (B; bottom panel) Surface SEM of a cross-section of MARY-X emboli displays canalis with microvilli (inset).

We next asked if the structural features, specifically the canalis, of the *in vitro *MARY-X spheroid recapitulated those of the *in vivo *MARY-X tumor cell emboli to further confirm the link between architectural features and metastatic efficiency. MARY-X form tight, compact aggregates of cells which manifest as spontaneously-formed spheroids *in vitro *and lymphovascular emboli *in vivo *(Figure 8A and 8B; top and middle panels). These human IBC cells (MARY-X spheroids) when injected into immune compromised mice will form complex primary tumors where the lymphovascular emboli are nestled within the murine surrounding stroma (Figure 8B; top panel). Upon malignant progression, pulmonary metastases will form within the pulmonary vessel network as simple (Figure 8B; middle panel, inset) or complex pulmonary metastases (Figure 8B; middle panel) comparable to the primary tumor (Figure 8B; top panel). Surface SEM analysis of a cross-section of MARY-X tumor emboli, like the *in vitro *MARY-X spheroid, reveals tortuous and highly bifurcating canalis (Figure 8A and 8B; lower panels). At higher magnification, the interior of the canalis of the tumor embolus is coated with microvilli (Figure 8B; lower panel, inset). Like the spontaneously formed *in vitro *MARY-X spheroid, the *in vivo *MARY-X tumor cell embolus displays unique architectural features. These data show that structural integrity of the malignant IBC is maintained throughout metastatic progression and most importantly, this makes MARY-X an *in vitro *model with tractable *in vivo *applications.

## Conclusions

A limitation in our understanding of epithelial tissue and tumors that are derived from them is that most insight into the formation, maintenance, function and pathology of epithelial tissue has depended on the analysis of traditional 2D cell tissue culture. This approach has been particularly useful in cancer research to determine drug response and toxicity in carcinomas. However, the results of a number of studies comparing the response of cells to drugs are often different when cells are cultured in 2D due to the failure to recapitulate the native 3D *in vivo *state [27,30]. In addition, 2D cultures differ significantly in morphology, signaling and differentiation. *In vitro *3D models (e.g. explants and traditional 2D cultures in matrix scaffolds) have been developed as an intermediate between the 2D cultures and animal models to better understand *in vivo *3D physiological conditions/environment [29,31,32]. These *in vitro *3D models offer expeditious assessment of drug response and toxicity and more closely resemble *in vivo *morphology and intracellular signaling [30]. Studies using 3D models appear to better correlate with drug response found *in vivo *[33]. However these models show significant variability of *in vivo *conditions and offer only short term *in vitro *conditions [30]. The most significant difficulty is the inability of these *in vitro *3D models to fully mimic the defined *in vivo *3D orientation, of either normal (i.e. polarity; distinct structural architecture) or aberrant (loss of structural integrity) epithelial cells, that dictates intracellular, intercellular and cell extracellular matrix (ECM) signaling [33].

Therefore, presently, the *in vitro *study of metastatic progression using 2D cultures has limited bearing on the *in vivo *situation. Even when the 3D architecture is recreated in the *in vitro *model or artificial soft agar scaffolds, the multicellular spheroids are dissimilar to micrometastases [30,33]. However, the MARY-X model is similar in that it grows as spontaneous, tight multicellular spheroids *in vitro *and as lymphovascular emboli *in vivo*, both displaying the same highly organized structural architecture. This true recapitulation provides us with a 3D *in vitro *model with tractable *in vivo *applications. The MARY-X model will allow us to manipulate these spheroids in culture and identify key gene products that convert IBC architecture to a more indolent carcinoma and determine if this affects metastatic behavior *in vivo*. Our study offers an alternative perspective as to what constitutes an aggressive carcinoma with a poor clinical outcome. Understanding carcinomas such as IBC that have a "gain in function" i.e. cellular organization could offer new strategies and approaches in treatment.

## Abbreviations

AJ: adherens junction; DM: desmosome; IBC: inflammatory breast cancer; SEM: scanning electron microscopy; TEM: transmission electron microscopy; TJ: tight junction.

## Competing interests

The authors declare that they have no competing interests.

## Authors' contributions

JM performed the electron microscopy analysis and data collection. MLA conceived of and designed the study, performed the immunofluorescence experiments and drafted the manuscript. All authors have read and approved the final manuscript.

## Authors' informations

MLA co-established the IBC xenograft, MARY-X, in 1998 during her postdoctoral fellowship. MARY-X was the first of only two IBC xenografts to be established. MARY-X has the distinction of exhibiting the signature phenotype of IBC that of extensive lymphovascular invasion *in situ *of the tumor cell emboli.

## Pre-publication history

The pre-publication history for this paper can be accessed here:

http://www.biomedcentral.com/1471-2407/9/462/prepub
